# Bioconversion of Used Transformer Oil into Polyhydroxyalkanoates by *Acinetobacter* sp. Strain AAAID-1.5

**DOI:** 10.3390/polym15010097

**Published:** 2022-12-26

**Authors:** Shehu Idris, Rashidah Abdul Rahim, Ahmad Nazri Saidin, Amirul Al-Ashraf Abdullah

**Affiliations:** 1 School of Biological Sciences, Universiti Sains Malaysia, Gelugor 11800, Malaysia; 2 Department of Microbiology, Kaduna State University, P.M.B. 2339, Kaduna 800283, Nigeria; 3 TNB Research Sdn Bhd, Kajang 43000, Malaysia; 4 Centre for Chemical Biology, Universiti Sains Malaysia, Bayan Lepas 11900, Malaysia

**Keywords:** bioconversion, used transformer oil, polyhydroxyalkanoates, *Acinetobacter* sp., bioplastics

## Abstract

In this research, the utilisation of used transformer oil (UTO) as carbon feedstock for the production of polyhydroxyalkanoate (PHA) was targeted; with a view to reducing the environmental challenges associated with the disposal of the used oil and provision of an alternative to non-biodegradable synthetic plastic. *Acinetobacter* sp. strain AAAID-1.5 is a PHA-producing bacterium recently isolated from a soil sample collected in Penang, Malaysia. The PHA-producing capability of this bacterium was assessed through laboratory experiments in a shake flask biosynthesis under controlled culture conditions. The effect of some biosynthesis factors on growth and polyhydroxyalkanoate (PHA) accumulation was also investigated, the structural composition of the PHA produced by the organism was established, and the characteristics of the polymer were determined using standard analytical methods. The results indicated that the bacteria could effectively utilise UTO and produce PHA up to 34% of its cell dry weight. Analysis of the effect of some biosynthesis factors revealed that the concentration of carbon substrate, incubation time, the concentration of yeast extract and utilisation of additional carbon substrates could influence the growth and polymer accumulation in the test organism. Manipulation of culture conditions resulted in an enhanced accumulation of the PHA. The data obtained from GC-MS and NMR analyses indicated that the PHA produced might have been composed of 3-hydroxyoctadecanoate and 3-hydroxyhexadecanoate as the major monomers. The physicochemical analysis of a sample of the polymer revealed an amorphous elastomer with average molecular weight and polydispersity index (PDI) of 110 kDa and 2.01, respectively. The melting and thermal degradation temperatures were 88 °C and 268 °C, respectively. The findings of this work indicated that used transformer oil could be used as an alternative carbon substrate for PHA biosynthesis. Also, *Acinetobacter* sp. strain AAAID-1.5 could serve as an effective agent in the bioconversion of waste oils, especially UTO, to produce biodegradable plastics. These may undoubtedly provide a foundation for further exploration of UTO as an alternative carbon substrate in the biosynthesis of specific polyhydroxyalkanoates.

## 1. Introduction

Synthetic plastics have become important commodities that have improved the quality of human life, replacing packaging materials like glass and paper [1]. The interest in the development of biodegradable plastics is largely generated from the problems associated with conventional plastics in a global environment [2]. Typical petroleum-derived plastics are non-biodegradable and mostly gather or aggregate around our environment, a problem that calls for great concern among communities, waste management agencies, and policymakers. Undoubtedly, plastic wastes pose a serious problem to landscape, marine animals, and wildlife and have since become an “environmental eye sore” [3]. Managing plastics wastes has become a global concern. Although it is hard to completely stop the use of petroleum-based plastics owing to their versatile utility, it is possible to substitute or reduce their usage with better alternatives by promoting the production and application of biodegradable polymers that have similar material properties [4]. Predictably, the plastics industry may witness a paradigm shift in the current century; this may ultimately bring about a change from an all-petroleum-based industrial economy to one that encompasses a relatively broader base of materials that include but not limited to fermentation byproducts [5]. The over-dependence on conventional plastics brings about waste accumulation and greenhouse gas emissions.

Consequently, recent technologies are directed towards developing bio-green materials that exert insignificant environmental side effects [6]. Petroleum-based mineral oils have been used in electrical transformers, primarily for insulating purposes. The oil serves the function of cooling the transformers. However, the long-term usage of transformer oil results in changes in its physical and chemical characteristics, which makes it unfit for cooling and insulating purposes. Thus, after being used up, the disposal of used transformer oil (UTO) from electrical power stations, as well as a large number of electrical transformers located in populated areas and shopping centres throughout the world, is becoming increasingly complex; this is so because it could contaminate waterways and soil if serious spills happen, this problem necessitates the need for providing an immediate solution [7]. Coincidentally, there is a continuous search for cheaper substrates to produce biodegradable plastics to reduce the high production cost that remains a big challenge to commercialising these eco-friendly alternative products. Consequently, several attempts are being made to produce bioplastics using waste oils. For instance, waste glycerol [8,9], palm oil [10], crude glycerol [11] and waste frying oil [12].

Polyhydroxyalkanoates (PHAs) represent a versatile group of prokaryotic reserve materials that display high potential for application in numerous fields of the plastic market, partly due to their plastic-like properties [13]. PHA are polyesters synthesised and accumulated by a number of taxonomically different microorganisms under nutrient-limited conditions, particularly when a carbon source is readily available. These biomolecules serve as carbon and energy storage materials; their presence has also been established to enhance resistance to various stress conditions [14]. For instance, recent research demonstrated that the biological role of PHAs goes beyond their storage function since their presence in cytoplasm increases the stress resistance of microbes [15]. PHAs have recently gained so much attention, especially in research institutions and industry. Undoubtedly, they are valuable materials with unique and desirable features; these important biological molecules attract attention as “green” alternatives to petrochemical plastics. However, The major disadvantage of these important biopolymers is their high production cost [1]. PHAs are mostly classified based on the number of carbon atoms in their respective monomers. Short chain length polyhydroxyalkanoates (*scl*-PHAs) have 3-5 carbon, medium chain length polyhydroxyalkanoates (*mcl*-PHAs) have 6-14 carbon, while the long chain length polyhydroxyalkanoates (lcl-PHAs) have 15 and above carbon atoms [3,16,17,18]. Among these classes of PHAs, *lcl*-PHAs is relatively the least explored, and this is evident in the limited number of published works on this important class of PHAs. In comparison with the short-chain-length PHAs, the medium-chain-length PHAs (*mcl*-PHAs) are relatively less abundant and frequently produced by bacterial species belonging to the genus *Pseudomonas* [19].

PHA-accumulating microorganisms can be isolated from diverse ecological niches like water sediments, sludge, rhizosphere, marine region, and coastal water body sediments [20]. Such habitats are often rich in organic nutrients and poor in other nutrients to meet the metabolic requirements of the starving microbial population, especially PHA-accumulating ones [21]. It has been established through extensive research that several PHA-producing microbes synthesise PHA toward the end of the log phase of their growth cycle [4]. It is also found that in later stages of their life cycle, they often use it as a carbonosomes [22]. Evidently, the presence of PHA inclusions within the microbial cytosol has served and will continue to serve as a chemotaxonomic signature for detecting various microbial isolates [4,23]. Numerous screening procedures have been developed for the detection of microorganisms that accumulate PHAs. Staining techniques involving the use of Nile red [24], Sudan black [25], and Nile blue [26] have, over the years, been used. Although these staining techniques cannot distinguish PHA inclusions from other lipids inclusion, a number of PHA-producing organisms have been successfully detected using this method, especially when complemented with other methods for confirmation [27]. Although analytical and molecular approaches are, in most cases, relatively time-consuming; and, therefore, difficult to apply in throughput screening, they are considered more reliable.

Despite the fact that PHA and other biodegradable polymers offer numerous environmental advantages such as composability, biodegradability, and biocompatibility, as well as the ease with which they become embedded in the natural carbon cycle, the main obstacle to commercialisation is their production cost. Consequently, the PHAs market penetration still lags behind the high expectations of the scientific community [28]. However, there is no doubt that PHAs biosynthesis and its related technologies are creating an industrial value chain ranging from materials, fermentation, and energy to medical fields [29]. PHAs can be applied in a number of ways that include, among others: packaging films, bags and containers, feminine hygiene products, surgical pins, sutures, swabs, wound dressing and staples, biodegradable carriers of drugs, medicines, insecticides, herbicides, or fertilisers, especially for long term dosage, replacements of bones and plates. In addition, PHAs are also applicable as starting materials for chiral compounds [30]. The relatively high production cost and the public awareness of the negative environmental impact of fluid mineral fuels-related products have been part of the driving force toward the search for novel raw materials with a ‘green agenda’ [31]. It is based on this background that the attempt was made to convert the UTO into PHAs with a view to providing an alternative to non-biodegradable plastics as well as reducing the production cost of PHAs.

## 2. Materials and Methods

### 2.1. Isolation of Organism and Growth Conditions

The bacterium used in this work was isolated from a soil sample obtained around Sungai Pinang Malaysia (Lat. 5°8′57‴ Lon. 100°24′38‴) [32]. The mineral salt medium (MSM) used for enrichment cultivation contains gram per litre of K_2_HPO_4_ (5.8); KH_2_PO_4_ (3.7); (NH_4_)_2_SO_4_ (1.1); MgSO_4_·7H_2_O (0.2); bacteriological agar (15). Other components include 1.0 mL of micro-element solution containing of FeSO_4_·7H_2_O (2.78); CaCl_2_·2H_2_O (1.67); MnCl_2_·4H_2_O (1.98); CoSO_4_·7H_2_O (2.81); CuCl_2_·2H_2_O (0.17); ZnSO_4_·2H_2_O (0.29); in 0.1M, HCl [33]. The MSM was supplemented with 4%(*v*/*v*) emulsified UTO (The emulsification was done in 1:1 ratio with Tween 80) as the sole carbon source. The enrichment culture was set up by inoculating 1 g of the soil sample into a 250 mL conical flask containing 100 mL of the medium and incubated at 30 °C for 48 h with an agitation speed of 200 rpm. Serial dilutions of the enrichment culture were made, after which 0.1 mL aliquot was taken from a selected dilution tube and inoculated on MSM agar (containing UTO as the sole carbon source) using the spread plate technique. The plates were incubated at 30 °C for 3–5 days. Pure colonies were sub-cultured and maintained in 20% glycerol at −20 °C.

### 2.2. Screening for PHA Accumulation

The stock culture of the isolated bacteria was activated in 30 mL nutrient-rich broth (10 g/L peptone, 2 g/L yeast extract and 10 g/L Lab Lemco powder). It was incubated at 30 °C for 18 h with an agitation speed of 150 rpm. After that, the culture was serially diluted using distilled water. An aliquot was then inoculated on solid MSM containing five(5) µg/mL Nile red [24]. The plates were incubated for 48 h at 30 °C. Colonies that formed were replicated onto a fresh MSM agar plate. The original plates were then exposed to ultraviolet illumination (320 nm) to identify PHA-accumulating organisms. Colonies that exhibit pink fluorescence were tentatively considered PHAs producers. Fluorescence microscopy was also used to further identify the PHA-accumulating isolates; in this approach, about 1 mL of 72 h old culture grown in MSM was transferred into 2.5 mL Eppendorf tube; after which 50 μL of Nile red solution (5 μg/mL) and 0.5 mL distilled water were added to the cell suspension and vortex-mixed immediately. The cell suspension was kept at room temperature for an hour and then centrifuged at 1000 rpm for 5 min. The supernatant was discarded, and the pellet was washed twice with distilled water. Another 0.5 mL of distilled water was then added to the pellet and mixed. Subsequently, 10 μL of the stained cell suspension was placed onto a clean glass slide and covered with a glass slip. The edge of the glass slip was sealed using cutex. Finally, the prepared slides were observed using a fluorescence microscope (Olympus BX53, Olympus Optical Co., Ltd., Tokyo, Japan) equipped with an Olympus DP72 camera. The prepared slide was observed under X100 UV compatible objective to detect the presence of PHAs granules. The granules normally excite Nile red and produce red fluorescence [34].

#### PHA Granules Visualization

Visualisation of the PHAs granules within the bacterial cell was accomplished using transmission electron microscopy (TEM) of the thin section of the bacterial cells embedded in Spur’s resin. Prior to resin block preparation, the test isolate was grown in a PHA production medium for 72 h at 30 °C. The culture was then harvested, and the cell pellet was washed twice with distilled water and fixed with McDowell-Trump fixative for one hour [35]. The fixed cells were subsequently treated with 1%(*v*/*v*) Osmium tetroxide for 1hr and suspended in 3% agar, followed by sequential dehydration in ethanol [50%, 75%, 95%, 100% (*v*/*v*); 30min each and finally with 100% acetone for 10 min]. The dehydrated cells were then embedded in low-viscosity Spur’s resin and cured overnight at 60 °C in the oven [36]. Ultrathin sections (1 µm thickness) were cut using a microtome (Power Tome PC-RMC Product, Boeckeler Instrument Inc., Tucson, AZ, USA). The thin section was placed onto the copper grids and stained sequentially with uranyl acetate and lead citrate solution for 15 min. Lastly, the thin section was observed at an acceleration voltage of 120 kV in the transmission electron microscope (Philip CM 12/STEM and JLM-2000FX11).

### 2.3. PHA Biosynthesis in Shake Flask

The PHA production was accomplished in a 250 mL Erlenmeyer flask containing 50 mL MSM supplemented with 2%(*v*/*v*) used transformer oil. The pH of the medium was adjusted to 6.8. the culture was incubated for 72 h at 30 °C, 200 rpm. After incubation, the culture broth was then centrifuged at 10,000× *g* for 10min. The cells pellet obtained was washed twice and freeze-dried in a freeze drier machine (LABCONCO, Kansas, USA). The cell dry weight (CDW) in g/L was estimated using an established method as described elsewhere [37]. The PHA content was subsequently analysed using gas chromatography (GC). The GC analysis was achieved using GC system (Shimadzu 2014, Kyoto, Japan) equipped with Flame ionization detector (FID) and fused silica capillary column (SPB-1 30 m × 0.25 mm × 0.25 mm). A 2 µL of the methyl ester obtained via methanolysis of the freeze-dried cells was injected into the system. An initial column temperature of 50 °C which was ramped to a final temperature of 250 °C in a continuous step of 5 °C/min was adopted with a total analysis time was set at 26.33 min.

### 2.4. Effect of Some Biosynthesis Factors on Growth and PHA Accumulation

The effect of some variable biosynthesis factors, such as concentration of carbon source (UTO), incubation time, and concentration of yeast extract, on the cell’s growth and polymer accumulation was assessed. All the experiments were carried out in three replicates, from which mean values were calculated accordingly.

#### 2.4.1. Effect of Carbon Source Concentration

A varying concentration of waste transformer oil ranging from 0.5, 1.0, 1.5, 2.0, and 2.5%(*v*/*v*) was used in order to assess the effect of the carbon source concentration on the organism’s growth and polymer accumulation. In the experimental setup, a centrifuged and washed cell inoculum (0.06 g/L) from the nutrient-rich broth was cultured in 50 mL MSM at 30 °C for 72 h with an agitation speed of 200 rpm. At the end of incubation, the growth in terms of cell dry weight (CDW) in g/L was deduced from the standard growth curve after measurement of the optical density (OD) at 540 nm. The biomass was recovered by centrifugation and then freeze-dried. The PHA content was determined using GC analysis.

#### 2.4.2. Effect of Incubation Time

The MSM was inoculated with 0.06 g/L inoculum of the bacteria and incubated at 30 °C with an agitation speed of 200 rpm. As the biosynthesis progresses, the sampling was made at a regular time interval of 12 h within a period of 120 h. Growth was monitored using spectrophotometry, and the CDW was determined by comparing the OD reading with the standard curve. The biomass was recovered by centrifugation and freeze-dried; PHA content analysis was subsequently carried out using GC techniques.

#### 2.4.3. Effect of Yeast Extract Concentration

The effect of yeast extract on the growth and PHA accumulation of the bacteria was assessed in the basal MSM medium supplemented with yeast extract at varying concentrations (0.5, 1.0, 1.5, 2.0, and 2.5 g/L). An inoculum (0.06 g/L) was cultured in 50 mL of the medium at 30 °C for 72 h with an agitation speed of 200 rpm. The bacterial growth through comparison of the OD with the standard growth curve. PHA content was later determined via GC analysis.

#### 2.4.4. Effect of Co-Carbon Substrates

In order to explore the metabolic capability of the test organism in terms of PHA synthesis, the effect of the combination of the main carbon substrate (UTO) with other additional/co-carbon sources was assessed. A varying concentration of UTO was used with a fixed amount of either oleic acid or palm oil (PO). In each case, 0.06 g/L inoculum was cultured in 50 mL MSM at 30 °C for 72 h with an agitation speed of 200 rpm. Growth was monitored using spectrophotometry, and the cell dry weight was subsequently determined. The biomass was recovered and freeze-dried, after which the PHA content was determined using Gas chromatography of the derivatised methyl ester.

### 2.5. Analytical Methods

#### 2.5.1. Polymer Extraction

The PHA was extracted using the solvent extraction method. Briefly, 200 mL of chloroform (CHCl_3_) was added to 1g of the dried cells in a Schott Duran bottle. The bottle was then tightly covered, and the cell suspension was stirred continuously using a magnetic stirrer for 48 h at ambient temperature. The cell debris was filtered using Whatman’s number 1 filter, and the solvent containing dissolve polymer was then concentrated to a volume of 10–15 mL at 60 °C using a rotary evaporator (Eyela, N-1000, Tokyo, Japan). The polymer in the concentrated filtrate was precipitated/recrystallised by dropwise addition of the filtrate into ten (10) volumes of well-stirred, chilled methanol. The suspension of the polymer was allowed to settle, after which the methanol was decanted, and the traces of the methanol were allowed to evaporate, leaving the sticky polymer for purification and subsequent analysis. Purification was achieved by dissolving the recovered polymer in chloroform, followed by concentrating the solution and the precipitation as done during the initial extraction [38].

#### 2.5.2. GC/GC-MS Analysis

The PHAs quantification was carried out through GC analysis using caprylic methyl ester (CME) as an internal standard. About 20 mg of freeze-dried cells were weighed into screw-cap tubes and subjected to acid methanolysis at 100 °C for 140 min. The methanolysis solution contains 85% (*v*/*v*) methanol and 15% (*v*/*v*) sulphuric acid [39]. The derivatised methyl esters were subsequently analysed by GC (Shimadzu GC-2014, Kyoto Japan) equipped with a capillary column SPB-1 (30 m length, 0.25 mm internal diameter and 25 µm film thickness; Supelco, Bellefonte, PA, USA) connected to a flame ionisation detector (FID). Nitrogen gas was utilised as carrier gas. Sample injection was made by autoinjector (Shimadzu AOC-20i) connected to the GC system. The injector and detector temperatures were set at 260 and 280 °C, respectively. The column temperature was ramped up from 60 to 280 °C at 5 °C/min with a total analysis time of 26.33 min. Identification of the monomer components of the polymer was achieved by GC coupled with mass spectroscopy.

#### 2.5.3. FT-IR Analysis

The FT-IR analysis was carried out using Fourier-transform infrared spectroscope (FT-IR-8300, Shimadzu, Kyoto, Japan). The sample casting was done on KBr pellets; the infrared spectrum was recorded between 400 and 4500 1/cm and 45 scans [40].

#### 2.5.4. NMR Analysis

The PHA recovered from the test organism was subjected to both ^1^H and ^13^C NMR using an FTNMR spectrometer (Acend^TM^ 500; Bruker, Switzerland). were carried out. Briefly, 5 mg of the polymer was dissolved in 2 mL deuterated chloroform (CDCl_3_) containing 0.03% (*v*/*v*) tetramethylsilane (TMS) as a reference standard. The solution was then filtered using a polytetrafluoroethylene (PTFE) filter (11807–25; Sartorius, Goettingen, Germany). The ^1^H spectrum was acquired at 500 MHz, 25 °C, with a sampling pulse of 3sec against TMS [41]. The ^13^C spectrum was also measured at 125.7 MHz, 27 °C with a sampling pulse of 1seconds. Chemical shifts were referenced to the residual proton peak of the deuterated chloroform at 7.26 ppm and to the carbon peak at 77 ppm [42].

#### 2.5.5. Size Exclusion Chromatography (SEC)

The molecular weight (Mw) data of the polymer were obtained using size exclusion chromatography system (Lachrom Merck–Hitachi, Darmstadt, Germany) equipped with a refractive index detector. The sample solution (1% *w*/*v*) was prepared in chloroform and filtered through a 0.45 μm pore size Sartorius membranes filter. Approximately 20 μL was injected with the flow rate set at 1.0 mL/min. The columns used were placed in series with exclusion limits of 10^6^, 10^5^, 10^4^ and 10^3^ Da. Chloroform with narrow polystyrene polydispersity (~1.1) was used for the calibration curve. The calculations were accomplished using clarity chromatography software version 8.0 [40].

#### 2.5.6. Thermogravimetry Analysis (TGA)

The thermogravimetry analysis was carried out using a TGA analyser (Netzsch TG 209, Selb, Germany). About 10 mg of pure polymer was used, and the sample was heated at a rate of 10 °C per min from 48.9 °C temperature to 898.6 °C in a nitrogen atmosphere [43].

#### 2.5.7. Differential Scanning Calorimetry (DSC)

About 15 mg of the pure polymer was measured and kept at 25 °C for 5 min. The sample was then heated to 125 °C through an incremental rate of 10 °C per minute to suppress the memory effect; the sample was kept at this temperature (125 °C) for 5 min and followed by cooling down to −100 °C at a rate of 20 °C per min and kept for 5 min before final heating to 350 °C at the rate of 10 °C per min. Melting temperature (*Tm*) was determined and recorded accordingly from endothermic peaks in the initial heating scan.

## 3. Results

### 3.1. Strain Characterisation and Screening for PHA Production

Nile red staining was applied in the microscopic observation for possible accumulation of PHA within the cell by the test organism. Brightly orange inclusions were observed under a fluorescent microscope; this indicates the possible presence of PHA granules. Further confirmation was made via transmission electron microscopy in which PHA granules were detected within the bacterial cells. This isolate was found to be a Gram-negative coccobacillus and designated as *Acinetobacter* sp. strain AAAID-1.5 with gene accession number MZ411700 [32]. The organism was used in the PHA production in shake flasks via a batch process biosynthesis with UTO as the sole carbon source.

### 3.2. PHA Biosynthesis in Shake Flask

The polymer biosynthesis in the shake flask experiment revealed that the bacterium had accumulated PHAs up to 34% of its dry weight (Table 1). From the table, it can also be seen that the polymer produced by the organism is composed of 3-hydroxyhexadecanoate and 3-hydroxyoctadecanoate monomers with mole fractions of 15 and 85 mol%, respectively.

### 3.3. Effect of Carbon Source Concentration

Varying concentrations of carbon source with a fixed (1.1 g/L) supply of nitrogen source was tested in shake flask biosynthesis. The influence of increasing concentration of the carbon source on the growth and PHA production is shown in Table 2. The results showed that the PHA concentration increased from 0.37 ± 0.03 g/L at 0.5%(*v*/*v*) concentration of the carbon source to the highest value of 0.72 ± 0.24 g/L at 2.0%(*v*/*v*) concentration of the same carbon source. Similarly, the growth showed an increasing trend with the increase in carbon source concentration. The molar fractions of the two monomeric components were roughly in a 1:4 ratio for 3HHD and 3HOD, respectively.

### 3.4. Effect of Incubation Time

The results obtained from this analysis are presented in Table 3. From the results, it is evident that PHA accumulation began in the early phase of the growth cycle. The PHA concentration almost doubled from 0.57 ± 0.05 g/L at 24 h to 0.96 ± 0.07 g/L at 48 h. The increase continued steadily and reached a maximum value of 1.19 ± 0.08 g/L at 72 h.

### 3.5. Effect of Yeast Extract

The effect of yeast extract as a supplement to the biosynthesis medium on bacterial growth and PHA accumulation was investigated. A control experiment was set without addition of yeast extract. The results are summarised in Table 4. It was observed that the addition of a lower concentration of yeast extract was more favourable to the polymer accumulation. The highest PHA concentration (1.25 ± 0.07 g/L) was observed at a yeast extract concentration of 1.0 g/L. Conversely, the biomass concentration increased steadily from 2.87 ± 0.04 g/L at a yeast extract concentration of 0.5 g/L to a maximum value of 3.46 ± 0.06 g/L at a concentration of 2.0 g/L. The 3HHD to 3HOD monomer ratio did not show wider variation across the range of the yeast extract concentration tested.

### 3.6. Effect of Additional/Co-carbon Substrates

The effect of Co-carbon substrates on growth and polymer accumulation was studied, in which the main carbon source (UTO) was combined with either of the two additional carbon substrates. The palm oil used as one of the additional substrates composed of 0.8% arachidic acid (C20:0), 0.3% lauric acid (C12:0), 0.2% linolenic acid (C18:3), 10.4% linoleic acid (C18:2), 47.7% oleic acid (C18:1), 3.7% stearic acid (C18:0), 36.3% palmitic acid (C16:0), 0.2% palmitoleic acid (C16:1) and 0.8% myristic acid (C14:0). The PHA accumulation by an organism during growth on different combination carbon sources (C1, C2, C3, and C4) at 30 °C for 72 h is presented in Table 5. The control experiment was set with the UTO as the sole carbon substrate. From the results, it was observed that the use of either of the two additional carbon substrates (palm oil and oleic acid) had a profound influence on both the PHA accumulation and the molar concentration of the monomeric components of the PHA produced. For instance, up to 3.02 g/L was recorded in the case of C4; this represents a 200% increase compared to the control experiment.

### 3.7. PHA Structure and Characterisation

**Figure 1:**
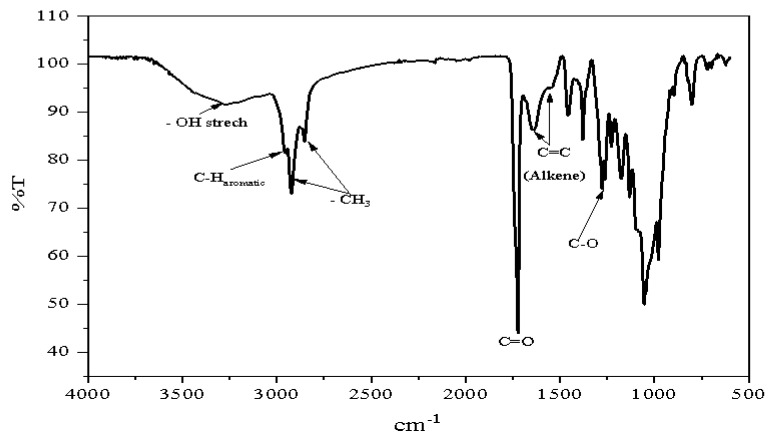
FT-IR spectrum of PHA produced *Acinetobacter* sp. strain AAAID-1.5 grown in MSM containing used transformer oil as carbon source.

The identification of monomer components of the PHA was achieved using GC-MS analysis. The spectral matching by the GC-MS system compared to the NIST08 standard reference library revealed the presence of 3-hydroxyhexadecanoate and 3-hydroxy-octadecanoate as the major monomer constituents. The FT-IR spectrum obtained from the analysis of the PHA extracted from the bacteria is shown in Figure 1; the sharp bending seen at 1722.67 cm^−1^ could be attributable to the carbonyl stretching of the polyester. The bends at 2922.82 and 2858.09 cm^−1^ signify the presence of the alkyl (-CH_3_) group, while the one at 1458.33 cm^−1^ could be due to asymmetric bending of -CH_3_ or -CH_2_ bending. The bending at 1397.01 cm^−1^ is considered to have stemmed from the symmetric bending of -CH_3_. The C-O stretching was represented by the bending at 1278.38 cm^−1^. Absorption spectra in the range of 800 to 1200 cm^−1^ signalled the existence of carbon-to-carbon stretches of an alkane group, while the band at about 721 cm^−1^ is typical of -CH_2_ side chains.

**Figure 2:**
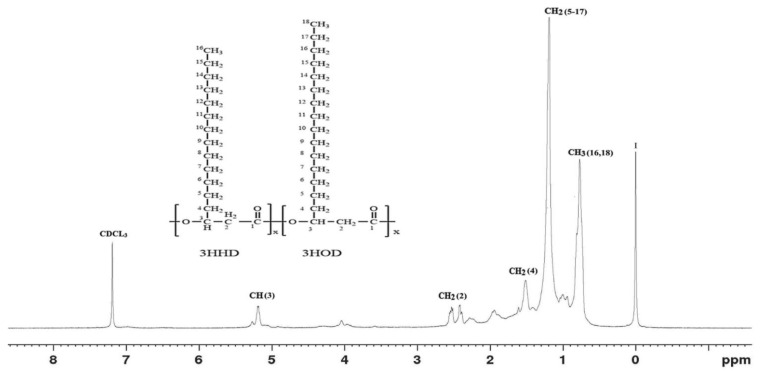
The ^1^H NMR spectrum of the PHA produced by *Acinetobacter* sp. strain AAAID-1.5 in a biosynthesis medium containing used transformer oil as a carbon source.

**Figure 3:**
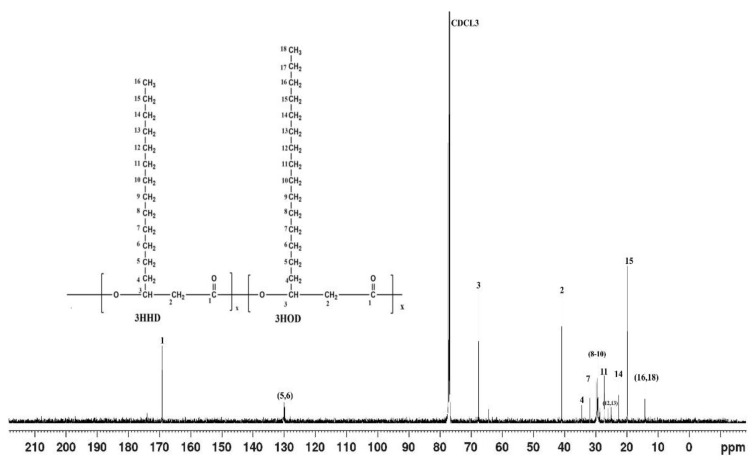
The ^13^C NMR spectrum of the PHA produced by *Acinetobacter* sp. strain AAAID-1.5 in a biosynthesis medium containing used transformer oil as a carbon source.

Figure 2 and Figure 3 show the spectra of the ^1^H and ^13^C NMR analyses. The chemical shifts were recorded in ppm relative to the signal of deuterated chloroform as an internal reference. In Figure 2, the singlet peak at δ = 5.2 ppm was assigned to the methine proton of the *β*-carbon, whereas the triplet peaks at δ = 2.6 ppm were deemed to be for methylene proton of the alpha carbon. The peak at 0.9 ppm was assigned to the methyl protons (-CH_3_) of the terminal carbon of the side chain, while those at 1.3 and 1.5 ppm were considered to have emanated from methylene protons, the peak at zero ppm was considered to be due to hydrogen atoms of the tetramethylsilane (I = TMS). The proposed PHA structure is shown in the figure.

In Figure 3, it can be observed from the chemical shift that the carbonyl groups (C=O) of the PHA backbone resonated at 169.14 ppm. The signals at 19.76 to 67.62 ppm correspond to methyl, methylene, and methine of the monomers.

In Table 6, some characteristics of the polymer produced by the bacterium at 4%(*v*/*v*) emulsified UTO, 30 °C, agitation speed of 200 rpm and incubation time of 72 h are presented. The results show that the polymer had an average molecular weight of 110.45 kDa and a polydispersity index of 2.01. The melting temperature was approximately 88 °C, while the highest degradation temperature was 268 °C.

## 4. Discussion

The successful isolation of *Acinetobacter* sp. strain AAAID-1.5 from a soil sample and its characterisation as a potent PHA-producing organism indicate that natural environments, especially soil, remain the reservoirs of effective biological agents that can be used to accomplish certain biotechnological processes. The ability of this bacterium to utilise the UTO as the sole carbon substrate to produce PHA has underscored its potential as a ‘seed’ for the valorisation of waste oil, particularly UTO, in bioplastic industries. A wide variety of wild-type bacteria, both Gram-negative and Gram-positive, such as *Pseudomonas*, *Cupriavidus*, *Bacillus* etc., have been implicated with the biosynthesis of PHA [44,45]. Similarly, *Acinetobacter* sp. was previously implicated with the ability to accumulate PHA, especially medium chain length PHAs [46]. Furthermore, a recent study described a species of *Acinetobacter* capable of utilising waste transformer oil as a sole carbon source [47,48]. The utilisation of waste streams for the biosynthesis of value-added products such as PHA is considered a cost-efficient strategy and can help address waste disposal problems [49,50]. Although some bacteria have been reported to synthesise PHA as much as 90% of their cell dry weight [6], A PHA accumulation of up to 34% of CDW was achieved by *Acinetobacter* sp. strain AAAID-1.5 in the shake flask experiment, and this indicated that the organism is capable of converting the UTO into PHA; to a reasonable extent that can be further explored for possible commercialisation. These findings substantiated a previous report in which a certain strain of *Acinetobacter* was reported to have accumulated PHA to the extent of approximately 25% CDW during biosynthesis in a medium containing various oil substrates [46]. Transformer oil and the wastes generated from there are a complex mixture of hydrocarbons, thus, the biosynthesis of PHA from such complex mixture was made possible probably due the ability of the bacterium to degrade the oil components using hydrolytic enzymes into chemical intermediates that can easily be channelled into the PHA biosynthetic pathway. For instance, *Acinetobacter* was reported to have the capacity of converting naphthalene (a major component of transformer oil) into acetyl-CoA via salicylic acid [51]. Oil hydrolysis during bacterial growth can also produce free fatty acids, that may undergo *β*-oxidation to generate precursors for PHA biosynthesis [52]. On the other hand, the limited growth shown by the organism in the first shake flask experiment presented in Table 1 where 2.1 g/L CDW was recorded; could be due to the presence of chemical components in the UTO that are resistant to microbial attack which might have caused toxic effect and slowed down the growth.

Regarding the effect of the carbon substrate concentration on bacterial growth and PHA accumulation, as presented in Table 2, it was observed that the results indicated that the increase in the concentration of the carbon source had a profound effect on the growth and PHA accumulation by the bacterium. PHA biosynthesis is normally induced by unbalanced growth conditions in the presence of excess carbon sources while essential nutrients such as nitrogen, oxygen, sulphur, and phosphorus are in limited supply [53]. The slight decline in PHA accumulation at carbon source concentration beyond 2%v/v might result from an inhibitory effect of some toxic chemical constituents of the carbon source that are hard to degrade. For instance, high concentrations of polyaromatic hydrocarbons (PAHs) may be toxic and can hinder metabolic activity. The ratio of molar concentration 3HHD to 3HOD was roughly 1:8 across the various concentrations tested, which could reflect the carbon composition of the bacterium’s degradation products derived from the UTO during biosynthesis, especially if fatty acids are the intermediate products. Occasionally, PHAs are synthesised with monomers equal in the number of carbon atoms to the fatty acid substrate [54].

The influence of incubation time on the PHA accumulation by the bacterium in this work showed an increasing trend until a maximum concentration of 1.19 ± 0.08 g/L was achieved at 72 h, as shown in Table 3. The decline observed beyond 72 h might be linked to the exhaustion of nutrients and accumulation of toxic metabolic waste in the late stationary phase of the growth cycle, as previously asserted elsewhere [55]. A significant accumulation of biosynthesis products during the late exponential phase is a typical feature of growth-linked biosynthesis [56,57]. Thus, the PHA accumulation pattern exhibited by *Acinetobacter* sp. strain AAAID-1.5 could be considered a growth-linked biosynthesis. Microbially produced polymers may vary as a function of the time at which the biomass is harvested [58]. Interestingly, the molar fractions of the monomer units of the PHA (3HHD and 3HOD) did not show a wider variation throughout the incubation time; this suggests that the molar concentration of the monomeric components of the polymer might be independent of the incubation time in this case.

With respect to the effect of yeast extract as a supplement to the biosynthesis medium on bacterial growth and PHA accumulation, the results, as presented in Table 4, the findings revealed that the addition of this supplement had improved the growth and PHA biosynthesis. This is evident when comparing the experimental control results with the other set of experiments in which the biosynthesis medium was supplemented with various concentrations of yeast extract. For instance, PHA accumulation increased from 0.72 g/L in the control experiment to 1.25 g/L in an experiment where yeast extract was added at a concentration of 1 g/L. this represents roughly a 42% increase. However, the ratio of the two monomer components of the polymer was roughly the same across different yeast extract concentration regimes. Yeast extract has been reported in a number of reports to have improved microbial growth and biosynthesis of some target products. Specifically, it was reported to have promoted PHA production and bacterial growth in recent studies by many authors [59,60,61]. The effect of yeast extract could be linked to its minerals and vitamin content which are critical to enzyme functioning and can ultimately facilitate the metabolic process.

The effect of additional carbon substrates in which either palm oil or oleic acid was added to the biosynthesis medium that already contained UTO showed that manipulating the carbon source could boost the PHA accumulation by the test organism. The fatty acid composition of the PO utilised as one of the additional carbon substrates is 0.8% arachidic acid (C20:0), 0.3% lauric acid (C12:0), 10.4% linoleic acid (C18:2), 0.2% linolenic acid (C18:3), 47.7% oleic acid (C18:1), 3.7% stearic acid (C18:0), 36.3% palmitic acid (C16:0), 0.2% palmitoleic acid (C16:1) and 0.8% myristic acid (C14:0). All the four different combinations (C1, C2, C3, and C4) of carbon substrate have produced a relative higher PHA content compared to the control experiment in which only UTO was used. This suggests that the organism might have exhibited a preference for carbon substrate. The free fatty acid is assumed to be more easily utilised by the bacterium than the complex mixture of hydrocarbons in UTO that may require degradation before usage. Palm oil and other organic oils, such as plant oils, are good feedstocks that can serve as excellent carbon sources for PHA biosynthesis [62]. Generally, oils are attractive carbon substrates for the sustainable production of PHAs, and they have been producing higher yields for both biomass and polymer synthesis [10]. PHA biosynthetic pathways are intricately linked to the organism’s central metabolic pathways such as glycolysis, de-novo fatty acids synthesis, *β*-oxidation, Krebs cycle, serine pathway, amino acid catabolism and Calvin cycle [63,64,65]. Therefore, the higher PHA content achieved upon the addition of the PO or oleic acid implied that the *β*-oxidation could have been a key pathway employed by *Acinetobacter* sp. strain AAAID-1.5. The improved PHA production upon the addition of oleic acid or PO has substantiated their influence on the biosynthesis mcl-PHAs in particular, as well as their attractive feature in PHA biosynthesis in general. For instance, PO was reported to have the ability to minimise the toxic effects of certain carbon substrates on bacterial cells [66]. Thus, the usage of PO in combination with UTO is assumed to have abated the suspected toxic effect of some constituents of the UTO.

With regards to the characterisation of the PHA in terms of physicochemical properties, the polymer produced appeared yellowish, amorphous, and sticky, with typical features of elastomeric polyester. The FT-IR spectrum recorded in the range of 600 to 4000 cm^−1^ showed spectral bending of a typical PHA that is comparable with the one previously reported by Cruz and his associates [67]. Interestingly, the sample of the PHA analysed had expressed substantial peaks similarity at various points compared to the spectrum obtained from *mcl*-PHA as reported elsewhere [41]. Furthermore, the data obtained from the GC-MS and NMR analyses of the synthesised polymer revealed that the PHA might have been composed of 3HHD and 3HOD as the major monomer constituents. This is comparable with a similar finding extensively described elsewhere [32]. In Table 6, the molecular weight and thermal properties of the polymer produced by *Acinetobacter* sp. strain AAAID-1.5 are presented. The number average molecular weight (M*_n_*) determined via Size exclusion chromatography in chloroform relative to polystyrene standard was 55.059 kDa. The average molecular weight and polydispersity index (PDI) were 110 kDa and 2.01 respectively. Whereas the melting temperature was about 88 °C and the highest degradation temperature was 268 °C. According to a previous report, *mcl*-PHA produced by *Acinetobacter* sp. ASC1 using crude glycerol as carbon source presents M*_n_* = 47 kDa, M*_w_* = 88 kDa and PDI = 1.9 [46]. The PHA produced in the present work presented a relatively higher M*_w,_* M*_n_* and PDI values, this might be due to longer alkyl side chain of the polymer produced in this study. Polydispersity index was determined by M*_w_/*M*_n_* as calculated in SEC. The PDI value (2.01) falls within the range of a typical *mcl*-PHA. For instance, it was reported that the PDI of *mcl*-PHA ranges between 1.1 to 6.0 [68]. The thermal temperature of *mcl*-PHA ranges between 30–80 °C [69]. However, a slightly higher value (88 °C) recorded in this work might be a result of factors that influence the thermal properties of the polyester. Temperature characteristics are the most variable features of PHAs, and they are critical to processing this important polymer. Such characteristic features determine the conditions under which the polymer can be processed and the properties of the resulting products [70]. Being a medium-chain length PHA which is elastomeric, the polymer produced in this work could be applied as biomedical materials, especially in the production of surgical mesh, sutures, vein valves, cardiovascular patches etc. Likewise, it could be applied in the production of packaging materials as well as in agriculture in the production of agricultural nets, mulch and in the controlled delivery of biofertilizer. Medium-chain length PHAs are preferred in biomedical applications because of their low crystallinity and are relatively more flexible in addition to being biocompatible [71,72].

## 5. Conclusions

The potential application of used transformer oil as feedstocks for PHA biosynthesis was established. Our study demonstrates that *mcl*-PHA polymers could be synthesised by a wild strain of *Acinetobacter* sp. utilising UTO as the sole carbon substrate. Furthermore, it was established that certain growth and biosynthesis parameters could influence the polymer accumulation in biosynthesis under control conditions. The study also provided insight into the potential of UTO as an alternative carbon feed in the production of biodegradable plastics using a biotechnological approach.

## Figures and Tables

**Table 1:** PHA biosynthesis by *Acinetobacter* sp. strain AAAID-1.5 using used transformer oil as the sole carbon source.

Biosynthesis Factors	CDW (g/L) ^a^	PHA Content (wt.%) ^b^	PHA (g/L)	RCDW (g/L)	Monomer Composition (mol%) ^b^	
					3HHD	3HOD
2% (*v*/*v*) UTO 200 rpm, 30 °C, 72 h	2.10 ± 0.04	34 ± 1	0.72 ± 0.24	1.38 ± 0.29	15 ± 1	85 ± 1

**Key**: CDW: Cell dry weight, RCDW = Residual cell dry weight, **^a^**: Cells were harvested after 72 h of incubation. Values are means ± SD of three experimental replications, **^b^**: Values are mean ± SD of three experimental replicates calculated from GC analysis, 3HHD: 3-hydroxyhexadecanoate, 3HOD: 3-hydroxyoctadecanoate.

**Table 2:** Effect of concentration of the carbon source on growth and PHA biosynthesis by *Acinetobacter* sp. strain AAAID-1.5.

Concentration of UTO (%*v*/*v*)	CDW (g/L) ^a^	PHA Content (wt.%) ^b^	PHA Concentration (g/L)	RCDW (g/L)	Monomer Composition (mol%) ^b^	
					3HHD	3HOD
0.5	0.94 ± 0.03	39 ± 2	0.37 ± 0.03	0.56 ± 0.01	17 ± 0	83 ± 0
1.0	1.43 ± 0.10	36 ± 1	0.51 ± 0.03	0.92 ± 0.07	14 ± 0	86 ± 0
1.5	1.58 ± 0.24	36 ± 2	0.56 ± 0.06	1.01 ± 0.18	15 ± 0	85 ± 0
2.0	2.10 ± 0.04	34 ± 4	0.72 ± 0.24	1.38 ± 0.29	15 ± 1	85 ± 1
2.5	2.11 ± 0.09	33 ± 6	0.69 ± 0.13	1.41 ± 0.15	20 ± 2	80 ± 2

**Key**: CDW: Cell dry weight, RCDW = Residual cell dry weight, **^a^**: Cells were harvested after 72 h of incubation. Values are means ± SD of three experimental replications, **^b^**: Values are mean ± SD of three experimental replicates calculated from GC analysis, 3HHD: 3-hydroxyhexadecanoate, 3HOD: 3-hydroxyoctadecanoate.

**Table 3:** Effect of incubation time on the growth and PHA biosynthesis by *Acinetobacter* sp. strain AAAID-1.5.

Incubation time (h)	CDW (g/L) ^a^	PHA Content (wt.%) ^b^	PHA Concentration (g/L)	RDCW (g/L)	Monomer Composition (mol%) ^b^	
					3HHD	3HOD
24	1.90 ± 0.09	30 ± 1	0.57 ± 0.05	1.33 ± 0.03	24 ± 0	76 ± 0
48	2.46 ± 0.04	39 ± 3	0.96 ± 0.07	1.49 ± 0.07	17 ± 3	83 ± 3
72	2.51 ± 0.08	48 ± 2	1.19 ± 0.08	1.32 ± 0.02	17 ± 3	83 ± 3
96	2.34 ± 0.07	50 ± 2	1.17 ± 0.08	1.17 ± 0.04	16 ± 5	84 ± 5
120	1.90 ± 0.09	50 ± 2	0.95 ± 0.06	0.95 ± 0.06	19 ± 0	81 ± 0

**Key**: CDW: Cell dry weight, RCDW = Residual cell dry weight, **^a^**: Cells were harvested after 72hr of incubation. Values are means ± SD of three experimental replications, **^b^**: Values are mean ± SD of three experimental replicates calculated from GC analysis, 3HHD: 3-hydroxyhexadecanoate, 3HOD: 3-hydroxyoctadecanoate.

**Table 4:** Effect of yeast extract concentration on the growth and PHA biosynthesis by *Acinetobacter* sp. strain AAAID-1.5.

Conc. of YE (g/L)	CDW (g/L) ^a^	PHA Content (wt.%) ^b^	PHA Concentration (g/L)	RCDW (g/L)	Monomer Composition (mol%) ^b^	
					3HHD	3HOD
0.5	2.87 ± 0.04	40 ± 2	1.15 ± 0.06	1.73 ± 0.05	17 ± 1	83 ± 1
1.0	3.36 ± 0.05	37 ± 2	1.25 ± 0.07	2.11 ± 0.11	19 ± 3	81 ± 3
1.5	3.63 ± 0.08	34 ± 1	1.23 ± 0.02	2.41 ± 0.11	17 ± 2	83 ± 2
2.0	3.46 ± 0.06	34 ± 4	1.17 ± 0.15	2.30 ± 0.10	18 ± 0	82 ± 0
2.5	3.42 ± 0.05	31 ± 1	1.08 ± 0.04	2.35 ± 0.10	18 ± 0	82 ± 0
Control	2.10 ± 0.04	34 ± 1	0.72 ± 0.24	1.38 ± 0.29	15 ± 1	85 ± 1

**Key**: YE: Yeast extract, CDW: Cell dry weight, RCDW = Residual cell dry weight, **^a^**: Cells were harvested after 72 h of incubation. Values are means ± SD of three experimental replications, **^b^**: Values are mean ± SD of three experimental replicates calculated from GC analysis, 3HHD: 3-hydroxyhexadecanoate, 3HOD: 3-hydroxyoctadecanoate.

**Table 5:** Effect of additional/Co-carbon substrates on the growth and PHA biosynthesis by *Acinetobacter* sp. strain AAAID-1.5.

Combination of Carbon Source	CDW (g/L) ^a^	PHA Content (wt.%) ^b^	PHA Concentration (g/L)	RCDW (g/L)	Monomer Composition (mol%) ^b^	
					3HHD	3HOD
C1	1.91 ± 0.05	45 ± 2	0.85 ± 0.03	1.06 ± 0.07	29 ± 2	71 ± 2
C2	2.10 ± 0.08	58 ± 7	1.21 ± 0.10	0.90 ± 0.17	33 ± 2	67 ± 2
C3	3.54 ± 0.13	67 ± 9	2.37 ± 0.39	1.17 ± 0.30	10 ± 1	90 ± 1
C4	3.85 ± 0.14	78 ± 4	3.02 ± 0.27	0.83 ± 0.14	09 ± 0	91 ± 0
Control	2.10 ± 0.04	34 ± 1	0.72 ± 0.24	1.38 ± 0.29	15 ± 1	85 ± 1

**Key**: CDW: Cell dry weight, RCDW = Residual cell dry weight, **^a^**: Cells were harvested after 72 h of incubation. Values are means ± SD of three experimental replications, **^b^**: Values are mean ± SD of three experimental replicates calculated from GC analysis, 3HHD: 3-hydroxyhexadecanoate, 3HOD: 3-hydroxyoctadecanoate, **C1**: 2% *v*/*v* UTO + 0.74% *v*/*v* palm oil, C2: 1% *v*/*v* UTO + 0.74 palm oil, C3: 2% *v*/*v* UTO + 0.74% *v*/*v* oleic acid, C4: 1% *v*/*v* UTO + 0.74% *v*/*v* oleic acid.

**Table 6:** Some characteristic features of the PHA produced by *Acinetobacter* sp. Strain AAAID-1.5.

Parameter	Value
M*_w_* (kDa)	110.45
M*_n_* (kDa)	55.059
PDI	2.01
*T*_m_ (°C)	88
*T_d_* (°C)	268

## Data Availability

Not applicable.
